# A new explanation for unexpected evolution in body size

**DOI:** 10.1371/journal.pbio.2001832

**Published:** 2017-02-22

**Authors:** Loeske E. B. Kruuk

**Affiliations:** Research School of Biology, The Australian National University, Canberra, Australia

## Abstract

Bigger is apparently frequently fitter, and body size is typically heritable, so why don’t animals in wild populations evolve towards larger sizes? Different explanations have been proposed for this apparent “paradox of stasis.” A new study of snow voles in the Swiss Alps finds higher survival in animals with larger body mass and heritability of body mass, but, surprisingly, a genetic decline in body mass is also indicated. The authors suggest a novel explanation for this observation: the appearance of positive phenotypic selection is driven by a confounding variable of the age at which a juvenile is measured, whereas the evolutionarily relevant selection actually acts negatively on mass via its association with development time. Thus, genes for larger mass are not actually “fitter” because they are associated with longer development times, and juvenile snow voles with longer development times run the risk of not completing development before the first winter snow. However, the genetic decline in body size is not apparent at the phenotypic level, presumably because of countervailing trends in environmental effects on the phenotype.

If bigger is better, will a population of animals evolve towards bigger body sizes, other things being equal? Not necessarily, and the reasons why are interesting. A recent study [1] of the dynamics of body size in snow voles sheds interesting light on this persistent problem [2,3] in evolutionary biology and proposes a novel explanation for some trickily counterintuitive observations. The arguments are appealing but complex, and the aim of this primer is to set out the background to and summarise the results of the study for a more general readership.

Artificial selection works by animal or plant breeders choosing which individuals they want to breed from and creating the next generation from these parents. Selection criteria are usually based on a particular phenotypic trait, such as size. The distribution of size amongst the selected parents will then differ from its distribution across the whole population. If size has a heritable genetic basis, the distribution of size in the offspring of the selected parents will differ from that which would have arisen had all individuals reproduced. Quantitative predictions follow on neatly: specifically, the change in the average value of a trait is simply the product of the strength of selection (how different the parents are from the whole population) and the heritability of the trait (a measure of the offsprings’ resemblance to their parents) [4]. This is the “breeder’s equation”; it is beguilingly simple, but, as I discuss below, it comes with a battery of assumptions [5].

In principle, natural selection works in the same way [6]. Some individuals have higher “fitness,” in that they contribute more than others to the gene pool of the next generation. If certain phenotypic traits have a consistent effect in determining these contributions—for example, through greater survival or reproduction—parents with these phenotypes will contribute more genes than others to the next generation. The association between phenotypic trait and fitness gives a quantitative measure of the strength of selection on the trait. If the phenotypic trait is heritable, the distribution of the relevant genes, and hence of the trait, should differ in the next generation—microevolution will have occurred.

Thanks to dedicated work in various field studies of wild species (typically animals, typically vertebrates), we can measure both selection and heritability in entirely wild, unmanaged populations [7]. Therefore, we should be able to predict responses to natural selection across generations and hence predict the temporal dynamics of important quantitative (continuous) traits. For example, studies of selection on size show, again and again, that bigger is better: numerous aspects of size have been shown to be positively associated with some component of fitness (e.g., [8]). Size traits are also the class of traits that are most consistently shown to be heritable (e.g., [9]). However, any initial excitement about applying the theory of artificial selection in the wild has been tempered by the fact that it flatly does not work: predictions for expected rates of microevolutionary response almost never fit with observed changes [2]. The general impression is of stasis, or even shrinkage, at microevolutionary time scales, and macroevolutionary rates of increase are also slower than might be expected [3]. So, evidence of positive selection on heritable size traits in wild populations rarely seems to generate trends that match the theoretical predictions of increasing size. Why this “paradox of stasis”?

Bonnet et al.’s [1] new analysis of the dynamics of body size in snow voles sheds interesting light on the problem. The authors present data from nearly a decade of careful monitoring of morphology and fitness in a high-altitude population living in scree slopes in the Swiss Alps. The analysis indicates positive directional selection on body mass: heavier individuals showed higher survival rates. Body mass was also heritable. However, there was little evidence for the expected increase in average body mass in the snow voles. So why are snow voles not getting larger at the rate predicted?

Obviously, evolution in response to natural selection in the wild is a more complicated process than one driven by animal breeders’ decisions. Several explanations for the “paradox of stasis” have been proposed, none of which are likely to be exclusive [10]. Most rely on dissecting out the contributions of genetics versus environment to phenotypes using the tools of quantitative genetics, the analysis of the genetic basis of continuous traits [4]. The approach provides an elegant and—assuming you have information on relatedness between individuals—straightforward means of quantifying key parameters. Analyses nowadays are typically based around an “animal model,” which in essence is just a glorified mixed model: an animal model estimates variance in additive genetic effects for each individual, with covariance structure for these effects defined by the relatedness of individuals (see [11] for an introduction). The simple premise is that the similarity (covariance) between two individuals in their underlying additive (heritable) genetic effects for a trait is determined firstly by their relatedness and secondly by the overall additive genetic variance for the trait in the population [12]. This is ultimately just an extension of simpler approaches that use the similarity between parents and offspring—or the similarity within groups of siblings—to estimate the heritability of a quantitative trait [4]. The models partition the total observed variance (diversity) in a trait between individuals into contributions from additive genetic effects versus other sources such as residual environmental variance. Usefully, the approach can be extended to two or more traits: by the same logic, the covariance between additive genetic effects for trait A in one individual and trait B in another individual depends on their relatedness and on the overall genetic covariance of the traits.

The snow vole study estimated covariances between body mass and relative fitness both at the phenotypic and, using an animal model analysis, at the genetic level [1]. In line with the estimate of positive selection, the overall phenotypic covariance between mass and fitness was positive, but—unexpectedly and counterintuitively—the additive genetic covariance had a substantial negative value. This conclusion held if fitness was estimated as lifetime reproductive success but was even clearer if just juvenile survival was considered.

Why is the genetic association between a trait and fitness informative? The additive genetic covariance between a trait and relative fitness gives an alternative estimate of the predicted response to directional selection on that trait. Named the Robertson–Price identity after its separate discoverers [13,14], this prediction is unfettered by many of the assumptions of the breeder’s equation. Most importantly, the breeder’s equation assumes either that there are no other correlated traits with causal effects on fitness, or that if there are, they are known and included in a multivariate analysis; in contrast, the genetic covariance reflects effects of all correlated traits that might be affecting fitness, whether or not they are known and measured [5]. It can also be used as a description of how the genetic component of a trait changes over time [5,15]. Applied retrospectively to the snow voles, the negative genetic covariance between mass and fitness therefore indicates genetic change towards *smaller*, not larger, sizes. A second line of evidence for this genetic decline comes from estimates of the trend in individuals’ “genetic merit” or “breeding values” from the animal model, a more complex analysis that requires careful treatment of the inherent statistical uncertainty [16]. This approach also indicated a genetic change towards smaller values, albeit with weaker statistical support.

The observation of negative genetic change therefore needed to be reconciled with the indication of positive selection on body size, and the authors tested several possible scenarios that might provide explanations. Firstly, size might be genetically tied to some other phenotypic trait in countervailing ways, such that an increase in body size reduced fitness through correlated changes in other traits—a classic evolutionary “trade-off” [17,18]. However, there were no indications that multivariate associations with other morphological traits generated opposing selection pressures in the snow vole population [1]. Nor was there any indication of a trade-off with parental fecundity, another hypothesis that proposes a general mechanism to explain stasis via parent–offspring conflict [19].

Alternatively, stasis in a heritable trait apparently under directional selection may occur when the association between the trait and fitness is not causal but driven by some externally determined variable—for example, environmental quality. Under this scenario, a favourable environment can generate larger body size and also higher performance, but there is no causal basis to the statistical association, just a joint correlation [20,21]. Failure to account for the relevant variable has the same effect as failure to include a “missing” but relevant phenotypic trait in a multivariate analysis. It derails the prediction of evolutionary response by the breeder’s equation, but not the prediction based on the genetic covariance [5,13].

Bonnet et al. [1] propose an explanation for the snow vole conundrum which I think can be viewed as a (potentially important) variation on the “missing trait” scenario. In this case, the process is dependent on not one but two “missing” traits. The first missing trait is the age at which a juvenile was caught and weighed in its first summer (juvenile age at measurement, termed “missing” because it could not be directly measured and so could not be included in the analyses; note that this is a finer-scale measure of age than the juvenile versus adult distinction which was included in the selection analyses in [1]). Size increases with a juvenile snow vole’s age (see Fig 3 in [1]), as does the individual’s probability of survival to the next census point: for example, animals first measured a month later have a month’s less risk of mortality. Variation between juveniles in their age at measurement therefore generates a positive association between size and survival (Fig 1), which will result in the appearance of a positive selection differential regardless of any causal effect of size on fitness.

**Fig 1:**
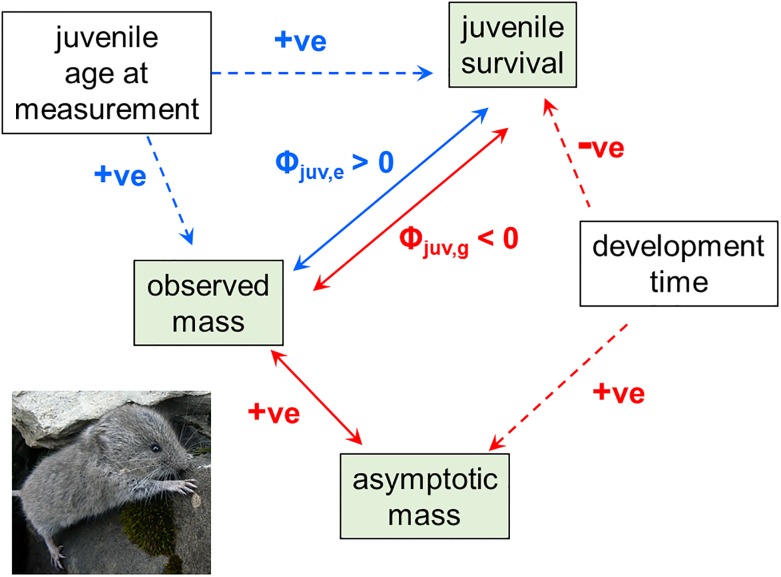
The hypothesised associations between traits and fitness in snow voles. “+ve” denotes a positive relationship; “-ve” denotes the negative relationship between development time and juvenile survival. Green boxes are traits that have been measured or (in the case of asymptotic mass) estimated; white boxes are “missing” traits. One-headed arrows imply direct effects; two-headed arrows represent covariance. Blue lines represent entirely nongenetic associations; red lines have some genetic basis. Solid lines represent estimated parameters; dashed lines represent inferred associations. Fitness is assessed by juvenile survival, relative to the population average. Φ_juv,e_ is the nongenetic covariance between juvenile survival and observed mass; Φ_juv,g_ is the additive genetic covariance (see Figure 3 in Bonnet et al. [1] for values). Photo copyright T. Bonnet.

The next piece of the puzzle involves an association between size and fitness driven by a second “missing” trait: development time, or the age at which an individual reaches its asymptotic body mass (Figure 3 in [1]). The snow vole population is situated high in the Swiss Alps, and there is strong selection for the pups born each year to reach their adult size before the arrival of the first winter snows. Thus, although individuals with longer development times will reach a higher asymptotic mass (development rate is roughly constant), they run the risk of higher mortality if snowfall is early, as it has been in the more recent years of the study. The net result is negative selection on development time and, by association, negative selection on asymptotic mass (Fig 1). The covariance between observed juvenile mass and estimated asymptotic mass is positive, and the implication is that the joint effects of the pathways by which genetic effects contribute to the covariance between observed size and survival result in a negative association (Fig 1). Two important conclusions emerge. First, the apparent positive selection is confounded by variation in age at sampling. How prevalent an issue this may be in other populations is an important question, not least because estimates of selection from wild populations are frequently based on measures taken on juveniles [19]. Secondly, the negative genetic covariance reflects selection driven by a different trait—development time—rather than by body size per se: on average, the costs of a longer development time outweigh the benefits of reaching a larger size. The net result is an adaptive genetic change towards smaller body size, but one that is not apparent at the phenotypic level, presumably because of counteracting effects of environmental trends affecting the phenotype via plasticity. These trends remain to be determined, but changing population density is suggested as one candidate.

Bonnet et al.’s hypothesis that the benefits of larger body size are negated by increased development time is a new addition to the list of possible explanations for stasis, and for contrasting patterns at phenotypic versus genetic levels. It will be interesting to see the idea tested in other studies and other taxa: ideally in systems in which it is possible to directly measure development time and size at known ages across the juvenile period so that the key “missing” traits are no longer missing, though of course that might be a tall order. However, the research also suggests several new avenues to explore. How will the processes described here interact with maternal effects, sex differences, litter size, or birth date? Can genomic data shed further light on the issue? What goes on beneath the snow? Not least, I hope the study helps secure the continuation of this valuable long-term study of a small mammal in Alpine conditions. To date, the most detailed individual-level studies of wild mammals have been of primates and ungulates [7]; smaller mammals provide welcome variety (and a more efficient generation time).

The paper is also valuable in demonstrating how effects of climate change on wild animal populations are not always driven by rising temperatures and their effects on spring timing of reproduction. Changes across the full annual cycle and in other aspects of climate such as patterns of snowfall may have profound ecological implications for wild mammals, especially small rodents (e.g., [22,23]). The general impact on natural selection of changing patterns of both precipitation and temperature thus remains a critical issue to be determined. Not least, in apparent contrast to the general evidence for positive selection on size, thermoregulatory arguments suggest that warming temperatures should favour smaller body sizes and hence that adaptation to climate change might involve declines in body size [24]. Overall, the long-term implications of climate change for selection and evolution in natural populations are still far from clear, and conclusive evidence of adaptive evolutionary responses to climate change remains surprisingly scarce [25].
